# Gelseminum

**Published:** 1877-08-20

**Authors:** 


### GELSEMINUM.

Dr. Adolphus says: A few words as to the mode of using the remedy in typhoid fever. There are many cases which commence with distressing nervousness, as it is called. These patients are very restless from the outset, tossing about the bed, having little sleep, are flighty at times from the beginning of the disease. As nightfall they are considerably worse, but perceptibly so every other day. These cases are well held in check by gelseminum. The excessive nervousness abates, restlessness subsides, sleep comes, the patient goes through the disease with far less wear and tear; in fact, recovers in real good order.

In all diseases where the temperature is very high and waste great, gelseminum acts well and does great good.

In all diseases where the motor part of the cord is greatly excited, gelseminum is a special remedy.

In all diseases where there is any exacerbation, gelseminum can be relied on as the great remedy.

The dose of gelseminum is of considerable moment as to the good it is capable of accomplishing. Driveling doses seldom fail to disappoint. The idea of two drops in a mug of cold water, and a teaspoonful doled out now and then, is a delusion—the dose must be decided. By decided I do not mean a reckless administration of a potent remedy. Some persons will not bear more than five drops at once before some drooping of the lids comes on, or a little double vision is manifested, while others will stand and really need ten, and others fifteen drops.

Some cases can bear five-drop doses every hour for four or five times; others will only tolerate a repetition in three or four hours. A little dropping of the lids, and even some slight dimness of vision, is requisite to obtain the best results of the remedy.

With these remarks, it is needless to extend this part of the subject any further.

Gelseminum has another very valuable property—its power to increase the pain-subduing powers of the anodynes. Thus, when united with belladonna or morphia, or both, the anodyne powers of these drugs are immensely increased, and much more so than one would suppose previous to practically experiencing the fact.

Another remarkable feature gelseminum possesses is, when combined with nux, the cambination has a remarkable tonic influence on the spinal system of nerves. In this way, many nervous affections are cured which neither alone would effect.

This combination often cures the colliquative diarrhœas of teething children, the neuralgia of nervous women; the pain and tormina of bladder and rectum diseases; arouses the exhausted nervous system in low fevers; checks the flow in excessive hemorrhage; relieves the pain of neuralgia. When a decided anodyne is needed, belladonna can be added with good results.—*Brief.*

